# Resistance mechanisms in melanoma to immuneoncologic therapy with checkpoint inhibitors

**DOI:** 10.20517/cdr.2019.28

**Published:** 2019-09-19

**Authors:** Sarah E. Fenton, Jeffrey A. Sosman, Sunandana Chandra

**Affiliations:** Division of Hematology Oncology, Northwestern University, Chicago, IL 60611, USA.

**Keywords:** Melanoma, checkpoint inhibitor, resistance, nonresponder, secondary resistance

## Abstract

Checkpoint inhibitors act by blocking physiologic mechanisms coopted by tumor cells to evade immune surveillance, restoring the immune system’s ability to identify and kill malignant cells. These therapies have dramatically improved outcomes in multiple tumor types with durable responses in many patients, leading to FDA approval first in advanced melanoma, then in many other malignancies. However, as experience with checkpoint inhibitors has grown, populations of patients who are primary nonresponders or develop secondary resistance have been the majority of cases, even in melanoma. Mechanisms of resistance include those inherent to the tumor microenvironment, the tumor cells themselves, and the function of the patient’s native immune cells. This review will discuss resistance to checkpoint inhibitors in melanoma as well as possible methods to restore sensitivity.

## Introduction

With 91,270 new cases diagnosed in 2018 and 9,320 fatalities, cutaneous melanoma is the fifth most common cancer in men and the sixth most common cancer in women in the United States. Additionally, due to lifestyle changes and other environmental factors, the incidence of melanoma continues to increase worldwide^[1-4]^. The majority of cases are diagnosed at an early stage and are managed with local excision. However, 30% of melanomas are diagnosed at a higher stage or progress to metastatic disease^[5]^. These patients require systemic therapy either to prevent recurrence or achieve disease control in hope of eradication. Prior to 2011, there was a paucity of efficacious therapies available for melanoma. Patients were treated with either dacarbazine or temozolomide, alkylating agents that inhibit DNA synthesis, or high dose interleukin-2 (IL-2), a recombinant analogue that acts to stimulate immune activity against the tumor. These treatments resulted in an overall response rate of 10%-20% with little effect on survival and durable long-term benefit in less than 10% of patients^[6-8]^. Fortunately, the landscape of systemic therapies for advanced melanoma has dramatically shifted with the introduction of checkpoint inhibitors and B-Raf V600 targeted therapies. Up to 45%-65% of patients treated with checkpoint inhibitors show an initial response^[9,10]^. Forty percent or more do not initially respond to treatment with checkpoint inhibitors due to a mechanism of primary resistance present before the initiation of therapy, these patients are classified as primary nonresponders. Additionally, at least 25%-30% of patients with metastatic melanoma that initially responded to treatment relapse over time through secondary mechanisms of resistance^[11]^. A pooled analysis of stage II and III trials of ipilimumab in advanced melanoma found that at year three approximately 20% of treated patients maintained disease control. If this positive response was observed, these patients had a high likelihood of maintaining disease control for up to ten years^[12]^. Although significant advances have been made with the introduction of checkpoint inhibitors, it is critical to increase our understanding of these mechanisms of resistance to improve the rates and duration of response. In this review we will discuss both primary and secondary mechanisms of resistance to checkpoint inhibitor therapy in melanoma, as well as treatments under development to restore sensitivity.

## Tumor recognition and antitumor immune response

Researchers such as Dr. William Coley in 1891 attempted to identify treatment strategies for malignant growths and noted that cancer patients occasionally underwent complete remission when they developed bacterial skin infections^[13]^. These observations suggested that activation of the immune system had unexpected activity not only against the bacteria but against the tumor cells themselves. In the 1950s through the 1970s, Ehrlich, Burnet and Thomas developed the theory of cancer immunosurveillance, suggesting that the immune system may be constantly identifying and targeting abnormal cells for lysis. However, when this immunity is weakened or overtaken by suppressive factors and cells, abnormal cancer cells may escape immune surveillance^[14,15]^. This careful balance is further defined by Dr. Schrieber in his theory of cancer immune-editing, where interaction between the host’s natural immunity and malignant cells exists in three phases: elimination, equilibrium, and escape^[16,17]^. Eventually these phases settle to where the immune system is no longer able to recognize the tumor and tumor cells are able to grow unchecked. Further supporting theses hypotheses, mouse studies of established tumors showed that inhibition of the T cell suppressive pathways resulted in tumor shrinkage^[18]^.

The elimination phase of the cancer immunosurveillance hypothesis is enacted when the immune system recognizes and eliminates malignant cells. One of the defining hallmarks of a cancer cell is its high mutation rate and the generation of abnormal proteins (peptides) from transcripts of mutated DNA, leading to neoantigen formation. Using innate mechanisms of immunosurveillance and response, tumor associated neoantigens can be taken up by antigen presenting cells, presented on their surface bound to MHC complexes and trafficked to lymph nodes where they bind to unique T cell receptors (TCRs) on naïve T cells. The binding of CD28 on the T cell and B7 on the antigen presenting cell leads to the stimulation and proliferation of the T cell and secretion of IL-2 to activate downstream immune regulators^[19,20]^. Tumor specific CD8+ T cells differentiate into effector T cells. They then traffic through the circulatory system to the tumor microenvironment where these effector T cells can identify and kill the tumor cells through perforin mediated lysis, granzyme mediated lysis, and the release of cytokines that induce apoptosis^[21,22]^. Ideally, these effector T cells then interact with CD4+ helper T cells and dendritic cells and differentiate into memory T cells, creating a durable mechanism of response to future tumor cells expressing the same neoantigen^[23]^.

The above pathways are utilized by the immune system to recognize and eliminate “non-self”, whether these abnormal cells are bacteria or tumor cells. In opposition to this, host cells have also evolved mechanisms to communicate their normalcy to immune cells. Unsurprisingly, tumor cells have in turn coopted these pathways to prevent immune cells from recognizing and eliminating malignant cells^[24]^. These changes characterize the equilibrium and escape phases of the cancer immunosurveillance hypothesis^[16,17]^. CTLA-4 is a receptor found on activated effector T cells and T regulatory cells (Tregs), which competes with CD28 to bind to B7 on the APC and prevent T cell activation^[25,26]^. Programed death receptor-1 (PD-1) and its ligand (PD-L1 and PD-L2) participate in another checkpoint mechanism for the immune system. PD-1 is expressed on the surface of T cells, monocytes, B cells, dendritic cells and tumor infiltrating lymphocytes (TILs) following initial activation. It is the activated T cell which has migrated into the tumor microenvironment that secretes γ-Interferon and induces PD-L1 expression on the tumor cell and surrounding inflammatory myeloid cells. When it binds PD-L1 on the surface of tumor or immune cells, immune tolerance is activated^[24,27]^. Tumor cells are able to coopt these and other pathways to prevent immune recognition and tumor cell targeting. Malignant cells have been shown to upregulate CTLA-4 and PD-L1 expression and downregulate presentation of antigens through genetic and epigenetic changes^[28-34]^. Tumor cells have also developed methods to alter signaling pathways and prevent cytotoxic T cells from inducing tumor cell death^[35]^. Using these adaptations, malignant cells are able to silence immune surveillance and grow unchecked.

## Checkpoint inhibitors and their role in melanoma

Six checkpoint inhibitors (ipilimumab, pembrolizumab, nivolumab, atezolizumab, durvalumab, and avelumab) are currently FDA approved in the treatment of several malignancies including metastatic melanoma, non-small cell lung cancer, renal cell carcinoma, Hodgkin’s lymphoma, urothelial cancers, head and neck squamous cell carcinomas, Merkel cell carcinomas and solid tumors with microsatellite instability or mismatch repair gene mutations. These agents act by targeting the CTLA-4 receptor, PD-1 or PD-L1, blocking tumor mediated immune suppression to allow immune recognition of abnormal proteins and immune mediated tumor cell death.

Ipilimumab, a fully human IgG1 monoclonal antibody, binds to CTLA-4 to block its engagement with B7, thereby preventing downregulation of the immune response. A phase III study of ipilimumab *vs.* a glycoprotein 100 melanoma-specific peptide vaccine in patients with previously treated metastatic or unresectable melanoma showed an overall response rate (ORR) of 10.9% with ipilimumab monotherapy (1.5% for the vaccine alone) and overall survival (OS) of 10.1 months with ipilimumab (6.4 months with the vaccine alone)^[36]^. Comparison of ipilimumab and dacarbazine dual therapy with dacarbazine monotherapy showed an overall survival of 11.2 months in the dual therapy group (*vs.* 9.1 months in the monotherapy group)^[37]^. Based on these trials, ipilimumab was approved by the FDA for the treatment of metastatic melanoma. When all phase II and III trials of ipilimumab in advanced melanoma were pooled for analysis median overall survival with treatment was 11.4 months. The survival curve plateaued at 22% at three years and continued at similar rates for up to 10 years^[12]^.

Another checkpoint inhibitor, nivolumab is a fully human monoclonal antibody of the IgG4 isotype that acts by binding to PD-1, preventing its interaction with its ligand PD-L1. A phase II trial comparing nivolumab with chemotherapy (institutional standard) in advanced melanoma found an overall response rate of 31.7% (compared to 10.7% with chemotherapy)^[38]^. In treatment naïve patients with metastatic melanoma, nivolumab resulted in a one-year overall survival of 72.9% (*vs.* 42.1% with dacarbazine), a median progression free survival (PFS) of 5.1 months (*vs.* 2.2 months with dacarbazine) and an overall response rate of 40% (*vs.* 13.9% with dacarbazine)^[39]^. Combination trials of ipilimumab with nivolumab resulted in response rates of 57.6% (95%CI: 52-63) and a median PFS of 11.5 months (95%CI: 8.9-16.7) in the combination therapy group (*vs.* a RR of 43.7% (95%CI: 38.1-49.3) and OS of 9.6 months (95%CI: 4.3-9.5) with nivolumab alone and a RR of 19% (95%CI: 14.9-23.8) and OS of 2.9 months (95%CI: 2.8-3.4) with ipilimumab alone)^[10]^. Another trial by Wolchok *et al*.^[40,41]^ found that three-year PFS rates for combination nivolumab and ipilimumab were 39% (*vs.* 32% for nivolumab alone and 10% for ipilimumab alone) while overall survival rates were 58% (*vs.* 52% for nivolumab alone and 34% for ipilimumab alone). Based on these trials and others, nivolumab is FDA approved to treat metastatic melanoma as a monotherapy and in combination with ipilimumab.

Another checkpoint inhibitor used in advanced melanoma is pembrolizumab, a fully human monoclonal IgG4 antibody against PD-1. A study of treatment naïve patients with metastatic melanoma found that pembrolizumab therapy resulted in a longer 3-year PFS compared to ipilimumab (31% *vs.* 14%). At 4 years overall response rate was 42% for pembrolizumab *vs.* 17% for ipilimumab alone. Rates of overall survival at 4 years were also increased with pembrolizumab therapy (41.7%) *vs.* ipilimumab treatment (34.1%)^[11,42]^. Based on these results, pembrolizumab is also FDA approved in the treatment of metastatic melanoma.

More recently, compelling data has been published regarding immunotherapy as an adjuvant treatment in stage III or IV melanoma after resection. Adjuvant ipilimumab therapy increased median recurrence free survival to 26.1 months (95%CI: 19.3-39.3) compared to 17.1 months (95%CI: 13.4-21.6) with placebo^[43]^. Rates of recurrence free survival at one year with pembrolizumab monotherapy were 75.4% (95%CI: 71.3-78.9) *vs.* 61% (95%CI: 56.5-65.1) with placebo^[44]^. Finally, nivolumab adjuvant therapy was found to be superior to ipilimumab monotherapy, with a one-year recurrence free survival rate of 70.5% (95%CI: 66.1-74.5) *vs.* 60.8% (95%CI: 56-65.2) with ipilimumab treatment^[45]^. Based on these trials, adjuvant checkpoint inhibitor monotherapy with ipilimumab, nivolumab or pembrolizumab has been approved by the FDA.

## Rates of resistance with checkpoint inhibitors

Two of the most revolutionary contributions of checkpoint inhibitors in the treatment of metastatic melanoma are the high rates of initial response and the ability to induce durable responses in a subset of patients. Unfortunately, rates of primary and secondary resistance remain high. Approximately 45%-60% of untreated metastatic melanoma patients treated with PD-1 inhibitors have an initial response, but by three years 43% of patients will have developed resistance and relapsed with fatal results^[10,39,46,47]^. Over 80% of melanoma patients treated with CTLA-4 inhibitor monotherapy have primary resistance^[36,37]^. In the KEYNOTE-001 trial of pembrolizumab, 25% of patients with an initial response were found to have progression of disease within a median time period of 21 months^[9]^. Another study with pembrolizumab found an initial response rate of 35%-40%, this had decreased to 33% overall survival at 3 years^[48]^. Due to the high rates of primary and secondary resistance, considerable work has been done to understand changes in signaling pathways, immune regulation and the tumor microenvironment to improve response rates and durability after treatment with a checkpoint inhibitor.

## Mechanisms of primary resistance intrinsic to the tumor cell

As noted above, between 40%-65% of metastatic melanoma patients do not show an initial response to checkpoint inhibitor mono- or combination therapy and so are categorized as primary nonresponders^[10,39,46,47,49-51]^. Many of these mechanisms of primary resistance result from abnormal interactions between the tumor and immune cells, preventing a successful anti-tumor immune response^[22,52,53]^. For example, lack of expression or presentation of altered antigenic peptides (neoantigens) due to low tumor mutational burden or defects in the antigen presenting machinery reduces recognition of abnormal malignant cells by immune cells^[52,54]^. Loss of function of transporters associated with antigen processing (TAP) proteins, β-2 microglobulin or silenced HLA expression all prevent the processing and trafficking of antigens to the cell surface^[55,56]^. β-2 microglobulin is a component of HLA class 1, when expression of this protein is lost HLA expression and antigen presentation are reduced due to inappropriate folding and transport of the HLA complex to the cell surface^[29,57,58]^. Downregulation of HLA on the tumor cell surface prevents immune mediated tumor cell recognition and killing^[59]^. To date, the tumor types successfully treated with checkpoint inhibitors such as melanoma, bladder cancer, lung cancer and solid tumors with microsatellite instability tend to have higher levels of tumor mutational burden and neoantigen formation^[19,60-63]^. Increased mutational load is associated with an increase in neoantigens with increased response to checkpoint inhibitors, but only if there is an associated increase in CD8+ T cell activation and infiltration^[60,64-66]^. Antigens presented on the tumor surface can be derived from viral antigens, non-mutated proteins where T-cell tolerance is incomplete (ex. MAGE-type antigens) and non-synonymous mutations that produce neoantigens^[19,67-69]^. Genetic and epigenetic changes in neoantigen expression can restrict T cells from recognizing abnormal antigens, inhibiting activation of the immune response^[21,28-31]^. Additionally, tumors with early expression of immunosuppressive pathways such as PD-L1 are protected from immune mediated recognition and elimination, resulting in an immune protected state. These cells are associated with higher rates of neoantigen production and increased susceptibility to immune attack once these immunosuppressive checkpoints are inhibited or reversed^[70]^. Finally, Giannakis *et al*.^[71]^ found mutations clustered in the TCR binding domain of the HLA protein in colon cancer, suggesting that successful cancer cells are clonally selected for their ability to escape T cell recognition through decreased antigen presentation. Hypermethylation of the HLA genes leading to decreased expression has also been identified in melanoma^[72-75]^.

Tumor cells can also bypass immune cell-mediated elimination through changes in apoptosis pathways activated by the immune effector cell^[76]^. For example, mutations in the interferon γ pathway can prevent apoptosis and tumor cell death^[55,56]^. Interferon γ signaling can be reduced through many pathways; however, one of the predominant mechanisms found in melanoma is loss of PTEN expression. Identified in 30% of melanomas, loss of PTEN expression resulted in increased PI3K signaling and decreased interferon γ and granzyme B expression by antigen specific T cells. Decreased interferon γ blocks recruitment of other immune cells and the induction of anti-proliferative and pro-apoptotic signaling in the tumor cells^[77-81]^. These changes are associated with poor response to PD-1 inhibitors^[77]^. A case report of a patient with a near complete response to PD-1 inhibition showed that one resistant lesion had biallelic loss of PTEN^[77]^. Interferon γ mediates some of its effect through JAK and STAT1 signaling. Through this same mechanism, loss of function mutations in JAK1 or JAK2 result in loss of interferon γ signaling, again preventing immune cell-mediated killing of the malignant cells^[27,29,82]^. In a small study of primary nonresponders, 75% of patients resistant to CTLA-4 inhibitors had a mutation in the interferon γ pathway or were found to have alternative changes leading to upregulation of negative regulators of the interferon γ pathway^[81]^. Deletion of interferon γ receptors, JAK1, JAK2 and STAT1 in mice with melanoma resulted in loss of PD-L1 expression and resistance to PD-1 inhibition^[83]^. Downregulation of other inducers of apoptosis such as caspase-8 and TRAIL can also allow evasion of immune-cell mediated cell death^[84,85]^. Acting through an inverse mechanism, increased expression of pro-survival genes such as BCL2 or BCL-XL in melanoma cells are associated with blockade of immune mediated apoptosis and cell killing^[76]^.

Expression of a specific subset of genes, called the innate anti-PD-1 resistance signature or IPRES, is associated with transformation of melanoma cells to a mesenchymal subtype, a reversion back to a more stem-cell like phenotype^[86,87]^. Upregulation of these genes may be caused by inflammation in the tumor microenvironment, and drives increased tumor plasticity. These genes are associated with resistance to checkpoint inhibitor therapy^[87,88]^. Upregulation of other specific genes associated with the epithelial to mesenchymal transition such as AXL, TWIST2, WNT5a, LOXL2, ROR2, TAGLN and FAP are also associated with primary resistance to PD-L1. This may be through the induction of a more plastic phenotype and upregulation of TNF-α^[87,89]^.

Thus far therapies have been developed to block two checkpoints for immune activation: CTLA4 and PD-1/PD-L1. However, other immune checkpoints exist and can be upregulated by tumor cells resulting in resistance to ipilimumab, nivolumab, and pembrolizumab therapy. These alternative checkpoints include the LAG-2 gene, TIM-3 (the T cell immunoreceptor that contains the immunoglobulin and ITIM domains), CD160, BTLA and V domain mediated immunoglobulin suppressors. These inhibitors of T cell activation and function have been studied in metastatic melanoma as well as head and neck, metastatic ovarian, prostate cancer and lung adenocarcinoma^[79,90-94]^.

## Mechanisms of primary resistance associated with the tumor microenvironment

As discussed above, there are several characteristics intrinsic to tumor cells that lead to primary resistance to checkpoint inhibitors. However, features of the tumor microenvironment and immune system are also associated with decreased or absent response to checkpoint inhibitor therapy^[95,96]^. Patients with notable responses to these drugs tend to have higher densities of CD8+ T-cells in the melanoma tumor prior to treatment^[97,98]^. Localization of immune cells within the tumor as opposed to at the tumor margin or complete absence from the tumor is associated with favorable results after checkpoint inhibitor exposure^[99]^. Response is also associated with the density of T cell and macrophage influx into the tumor microenvironment after treatment is initiated^[29,100-106]^. Lack of sufficient breadth or depth of T cells primed to recognize and react against specific tumor antigens results in poor immunogenicity after checkpoint inhibitor therapy^[52]^. In fact, the ability to recognize neoantigens as non-self is likely as important to the success of checkpoint inhibitor therapy that the actual number of mutations and neoantigens expressed on the cell surface^[107-109]^. Mechanisms that inhibit T cell proliferation and the priming of naïve T cells can result in a limited pool of available T cells and decreased antigen recognition^[101,110,111]^. Specifically, downregulation of MHC proteins on dendritic cells prevents antigen presentation and immune cell activation, limiting response to checkpoint inhibitors^[59,112-116]^. Dendritic cell function is primarily decreased in the tumor microenvironment through tumor mediated expression of IL-37b and STAT3 (which activates VEGF, IL-10 and TGF-β)^[117-121]^. Downregulation of immune cell recruitment through signals such as CXCR3 can also decrease T cell homing to the tumor site^[122]^.

Tumor cells can prevent T cells and other immune cells from entering the tumor microenvironment. Mechanisms of T cell exclusion include MAPK signaling, β-catenin stabilization, expression of a mesenchymal transcriptome and PD-L1 expression^[55,56]^. Upregulation of MAPK signaling increases VEGF and IL-8 production, inhibiting T cell recruitment and function^[123]^. Increased β-catenin signaling results in upregulation of WNT signaling and reduced dendritic cell and T cell recruitment, handicapping the ability of immune cells to act within the tumor microenvironment^[101,124,125]^. Melanoma tumors with increased infiltration by CD8+ T cells and low β-catenin signaling were associated with longer PFS^[125]^. Additionally, upregulation of EZH2 expression causes CXCL9 and CXCL10 downregulation, decreasing T cell recruitment to the tumor, and promotes the tumor cells to undergo transformation to a more mesenchymal phenotype^[126-132]^. Inhibition of this pathway is associated with increased immunogenicity and T cell infiltration in mouse melanoma tumors^[133]^.

Apart from lack of immune cell infiltration, the recruitment of regulatory or suppressive immune cells also determines refractoriness to immunotherapy. Tregs can suppress the function of other immune cells in the microenvironment through secretion of IL-6, IL-10, IL-35 and TGF-β, as well as through direct inhibitory contact with other T cells. This creates an immune tolerant environment through increased Treg recruitment and inhibition of T effector cell function^[52,134-138]^. Tumor associated macrophages express PD-L1 and B7-H4 to downregulate T cell responses^[139-142]^. The presence of myeloid derived suppressor cells (MDSCs) has also been associated with poor response to immunotherapy^[143-145]^. In fact, levels of MDSCs may be used as a predictive marker of response to ipilimumab therapy in metastatic melanoma^[143]^. These cells are recruited in response to IL-6, IL-10 and TGF-β secretion by the tumor cells to downregulate tumor antigen recognition, prevent T cell proliferation and inhibit cytotoxic T cell function^[137,138]^. Finally, cancer associated fibroblasts express TGF-β, reducing T cell infiltration into the tumor environment through a similar mechanism as Treg cells^[97,146-148]^.

Upregulation of Fas ligand on intratumoral blood vessels through VEGF-mediated proangiogenic signaling by the tumor is another mechanism that reduces immune cell trafficking into the tumor microenvironment^[149,150]^. The presence of the Fas ligand induces apoptosis of Fas+ CD8+ T cells when they come in contact with the pericyte wrapped around the blood vessel during migration. Tregs remain unaffected and traffic freely into the melanoma tumor. Upregulation of the Fas ligand is highly specific to blood vessels within the tumor, as it is often not seen in surrounding tissue^[151]^. Increased expression of the endothelin-B receptor may play a similar role, decreasing the recruitment of TILs^[152,153]^. Acting through a separate pathway, VEGF also upregulates NF-κB signaling, limiting dendritic cell maturation and antigen presentation^[154-156]^.

Due to the high metabolic demands of the malignant cells, the tumor microenvironment is an environment that may block adequate immune cell function. Tumor cells and T cells often compete for necessary metabolic substrates such as glucose, cholesterol, and specific amino acids such as arginine and glutamine^[35,157-159]^. Additionally, the tumor microenvironment tends to be highly acidic with increased concentration of tumor derived lactate and hypoxia from malignant cell consumption, reducing T cell function due to their dependence on aerobic glycolysis^[157,160-166]^. Unfortunately, Tregs are not as dependent on the presence of these factors and their function is likely less limited in the tumor microenvironment, adding to the overall immunosuppression^[21]^. Finally, hypoxia induces ATP release into the tumor microenvironment. Dephosphorylation of ATP to adenosine results in ligation of the adenosine with the A2A receptor on T effector cells, blocking their function^[161]^.

Expression of inflammatory signaling molecules such as TGF-β, indolamine-2,3-dioxygenase (IDO), IL-10, and arginase by stromal cells and leukocytes within the tumor also suppresses immune cell function^[167-171]^. IDO expression specifically catabolizes tryptophan to kynurenine to inhibit dendritic cell function, block T cell proliferation, and increase MDSC and Treg infiltration^[171-178]^.

Finally, as in most other areas of the body, the tumor microenvironment involves a microbial component. These microbes act through Toll like receptor signaling to alter the infiltration of monocytes, reducing immune responses and the efficacy of checkpoint inhibitors^[179,180]^. Additionally, microbial metabolism and fermentation of fibers generate butyrate in the tumor microenvironment. As cancer cells cannot use butyrate for energy, competition for resources between microbes and cancer cells may inhibit malignant growth. Butyrate can also act as a histone deacetylase inhibitor to promote apoptosis and inhibit proliferation^[181]^. Bifidobacterium in the microenvironment is associated with increased dendritic cell function and mouse models show inhibition of melanoma when Bifidobacterium is present^[182]^. Similar results have been seen with Bacteroides fragilis and Bacteroides thetaiotamicron in other models where cytotoxic T-cell function is augmented in the presence of these bacteria^[183-185]^. Although the above mechanisms may have an anticancer effect, researchers have also identified roles for the microbiota that may promote tumor growth including local inflammation and tissue damage. Extrapolating from microbe’s role during infection, it is also possible that bacteria in the microenvironment promote angiogenesis^[186]^. Additionally, upregulation of T helper cells in the microenvironment is associated with tumorigenesis in colorectal cancer^[187]^. More specifically, the presence of Fusobacterium nucleatum has been associated with silencing of cytotoxic T cells, upregulation of MDSCs and increased tumor associated macrophages in the microenvironment^[188,189]^.

## Mechanisms of secondary resistance

A significant proportion of melanoma patients treated with checkpoint inhibitors exhibits an initial response. Fortunately, for a subset of patients these responses are durable and long lasting. However, the majority of patients experience relapse and the development of secondary resistance mechanisms. The majority of these mechanisms are similar to those found in primary nonresponders. Exposure to checkpoint inhibitors drives selection for tumor cells that have developed escape mechanisms or may alter immune cell functioning to prevent successful malignant cell elimination. One primary mechanism of secondary resistance is downregulation of antigen presentation on the tumor cell surface^[28,32,33]^. This may be mediated by upregulation of interferon γ signaling after checkpoint inhibitor exposure^[190]^. A study of relapsed non-small cell lung tumors by Anagnostou *et al*.^[191]^ found that on average 7-18 potential neonatigens were lost after treatment with checkpoint mono- or combination therapy. These neoantigens were associated with stronger anti-tumor responses than those that were retained, suggesting selective immunoediting.

Specific mutations have also been identified in melanoma cells after the development of secondary resistance. A late relapse patient with metastatic melanoma resistant to CTLA-4 inhibition was found to have biallelic loss of β-2-microglobulin^[29,110]^. Zaretsky *et al*.^[29]^ also identified a patient with homozygous frameshift deletions of β-2-microglobulin that had developed resistance to PD-1 blockade^[28-30,192]^. Five patients treated with immunotherapy were found to have reduced MHC expression and were not recognized by effector T cells due to loss of β-2-microglobulin expression. Three of these patients were known to have normal β-2-microglobulin expression prior to treatment^[58]^. Acquired resistance to PD-1 inhibitors has also been associated with inactivating mutations in JAK1 and JAK2, leading to escape from interferon γ signaling and immune-mediated tumor cell death^[29,82]^. Four melanoma patients treated with pembrolizumab were found to have loss of function mutations in JAK1/2 in relapsed tumors^[29]^.

Similar to mechanisms of primary resistance, secondary processes are associated with changes in the balance between immune effector and repressor cells in the tumor microenvironment. Study of resistant melanomas suggest that they have reversed back to a T cell excluded state^[29]^. CTLA-4 and PD-1/PD-L1 inhibition is also associated with upregulation of other inhibitory pathways such as EZH2, LAG-3, TIM-3, TIGIT and VISTA, silencing immune activity through another mechanism^[79,93,177,193,194]^. Finally, VEGF is upregulated in relapsed patients, suggesting secondary resistance may also occur through Fas ligand and other VEGF-mediated mechanisms^[97]^.

## Mechanisms of immune cell exhaustion and lack of memory cell formation

Activation of the immune response is associated with upregulation of innate immune suppressors to prevent an uncontrolled immune reaction. Therefore, treatment with checkpoint inhibitors and persistent immune activation can lead to an exhausted T cell phenotype with upregulation of surface receptors such as LAG-3 and TIM-3 among others and ultimately result in treatment failure^[27,92,195-197]^.

Interestingly, expression of PD-L1 is associated with signaling to promote effector T cell exhaustion. Treatment with PD-1/PD-L1 inhibition may reverse this exhaustion phenotype and rescue T cell function, however this change is not associated with the expected next steps of memory T cell formation, limiting the duration of anti-tumor response^[23,102-105,198-200]^. CTLA-4 inhibition, on the other hand, is associated with expansion of effector T cells that express alternative immune checkpoints and result in further immunosuppression^[92,94,201]^.

Studies of gene expression profiles within tumors using single cell RNA sequencing technology confirm these findings. Lack of Tcf7 expression, a protein involved in the differentiation and persistence of memory T-cells, is associated with resistance to treatment with checkpoint inhibitors. Together this data suggests that the formation of memory T cells is a critical component of patient responses when treated with immunotherapy and failure of this pathway is associated with drug resistance^[202,203]^.

## Mechanisms of resistance associated with the patient

Interestingly, factors outside of the tumor cells themselves and the microenvironment that they thrive in have been associated with checkpoint inhibitor failure. A stool microbiome enriched with bacteria from the Raecalibacterium genus and other firmicutes is associated with increased progression free and overall survival after treatment with ipilimumab, as well as higher rates of immune mediated colitis^[204]^. Metastatic melanoma patients with higher gut microbiome diversity also have a higher density of intratumoral effector T cells after checkpoint inhibitor exposure^[204,205]^. Recent antibiotic use and associated reductions in gut microbiome diversity has been associated with resistance in renal cell carcinoma treatment with anti-PD-1, PD-L1 and combination therapies^[206]^. Although the exact mechanism underlying this association is unknown, it may be due to increased priming of dendritic cells with antigens that cross-react with tumor neoantigens^[182,207,208]^. The bulk of the research done on interactions between the gut microbiome and checkpoint inhibitors has been performed in mice. As this research is applied to patients and the human gut microbiome a more nuanced approach is likely required, as both the presence of melanoma and the use of checkpoint inhibitors is associated with shifts in the balance between different phylum of bacteria in the gut^[209,210]^. Whether these changes are a driver of resistance or the result of other mechanisms of resistance at play has not yet been established.

Finally, certain clinical findings are associated with primary nonresponse to checkpoint inhibitor therapy. An elevated serum neutrophil to lymphocyte ratio is associated with poor survival following checkpoint inhibitor therapy while an overall low neutrophil count and low LDH is associated with increased survival^[165,211]^. In another study of patients with metastatic melanoma treated with pembrolizumab, a high serum eosinophil and lymphocyte count and a low LDH was associated with increased survival^[164]^. High tumor burden at the time of treatment initiation is also associated with primary resistance^[64,212,213]^.

## Novel therapies to bypass resistance

Due to the high demand for effective systemic therapies for metastatic melanoma and recent advances with PD-1 and CTLA-4 inhibitors, significant work is ongoing to reduce rates of primary and secondary resistance to these drugs. The majority of these investigations focus on reversing specific mutations and pathways known to be associated with treatment failure. For example, JAK1/2 inhibitors are associated with overcoming resistance^[79]^. Alternatively, the introduction of agonists for proteins downstream of JAK1/2 such as STAT1 may also restore immune activity^[29,214,215]^. Anti-angiogenic drugs have been shown to improve lymphocyte trafficking and migration as well as reverse immunotherapy resistance^[77,216,217]^. VEGF inhibition in melanoma is associated with increased levels of TILs through increased CXCL10 and 11, inhibition of prostaglandin E2 also restores effector T cell infiltration^[218,219]^. Treatment with 5-azacitadine may reverse hypermethylation of MHC genes, restoring antigen presentation^[220-223]^. Adding Lag-3 inhibitors has been associated with tumor regression and increased survival in mice, the addition of a poxvirus to this regimen is also associated with improved responses in mice^[224,225]^. Dual blockade of TIM-3 and PD-L1 or CTLA-4 is associated with tumor response and increased T cell function^[226,227]^.

Beyond reversing specific pathways or mutations associated with checkpoint inhibitor failure, combination therapy with other drugs is being explored to alter more general changes to improve checkpoint inhibitor efficacy. Adding indoleamine inhibitors to treatment strategies could help relieve metabolic rate limiting steps in the tumor microenvironment^[21]^. Checkpoint inhibitor failure has been associated with the development of an exhausted immune phenotype; and adding histone deacetylase inhibitors, epigenetic modifying agents (ex. disease modifying anti-rheumatic drugs or DMARDs) or targeted therapies for the exhaustion pathways may prolong the response to immunotherapy and antigen presentation^[104,105,228]^. Finally, adding IDO inhibitors such as the peptide vaccine formulated against IDO, indoximod or epacadostat may restore the efficacy of immunotherapy^[229-231]^.

Clinicians and researchers are also improving the efficacy of checkpoint inhibitors by optimizing the immune response induced by PD-1 and CTLA-4 blockade. Essentially, these therapies are acting to turn a “cold” tumor microenvironment into a “hot” tumor microenvironment where the immune system is active against the malignant cells. Increasing the ability to upregulate effector T cells or downregulate Tregs would dramatically improve responses and decrease rates of resistance^[52,134-136]^. Combination therapies proposed to alter the interaction between the immune system and tumor include agents such as cyclophosphamide or oxaliplatin to increase the priming of effector T cells, PI3Kγ to inhibit myeloid suppressor cells, CSF-1R inhibitors to downregulate tumor associated macrophages and TGF-β inibitors to downregulate Tregs^[35,139-145,232-236]^. Recent work with single cell RNA sequencing methods have identified programs of gene expression within the tumor associated with poor response to checkpoint inhibitors. Further study of these gene expression patterns suggest that treatment with CDK4/6 inhibitors may shift the tumor cells to a less immune resistant state, increasing response to checkpoint blockade^[237]^. Priming MHC class II receptors could potentially increase CD4+ T cell activity, bypassing difficulties seen with MHC I inhibition in melanoma cells with β-2 microglobulin and other mutations^[238]^. The addition of cancer vaccines (with either peptide vaccines or with primed dendritic cell vaccines) or the additional of radiation therapy to increase neoantigen formation and recognition may augment T cell responses and tumor recognition^[35,239-242]^. Similarly, adding cytokines that stimulate the immune system such as IFN-a, IL-2, IL-12, IL-10 and the granulocyte-macrophage colony-stimulating factor (GM-CSF) to the treatment regimen or at high concentrations directly into the tumor microenvironment may also alter immune responses^[243]^.

Finally, adding probiotics to a patient’s treatment regimen may increase drug efficacy and treatment response. This has been successfully shown to improve responses in mouse models of melanoma^[182]^. However, this data remains controversial as recent studies have shown that melanoma patients tend to have a lower diversity within the gut microbiome than healthy patients and that probiotic use further decreased this diversity. These findings suggest that probiotic use may be more complex than previously understood and may not be as directly associated with improved outcomes^[210]^.

## Conclusion

Resulting in nearly 10,000 deaths per year, melanoma presents a significant challenge for cancer researchers and clinicians^[1]^. Fortunately, recent years have seen a revolution in melanoma therapy with the introduction of checkpoint inhibitors. Despite the significant improvement in outcomes, rates of primary resistance and relapse remain high in melanoma patients treated with checkpoint inhibitors. Therefore, diverse approaches detailed in this review are being pursued to improve clinical outcomes and their duration. These include novel treatment strategies that target mutations within the tumors themselves, mechanisms that inhibit an adequate immune response to tumor cells, changes within the tumor microenvironment and factors present in the patient to improve the efficacy of checkpoint inhibitor mono- and dual therapy.

## Declarations

### Copyright

© The Author(s) 2019.
